# The tumor area occupied by Tbet+ cells in deeply invading cervical cancer predicts clinical outcome

**DOI:** 10.1186/s12967-015-0664-0

**Published:** 2015-09-10

**Authors:** Arko Gorter, Frans Prins, Merel van Diepen, Simone Punt, Sjoerd H. van der Burg

**Affiliations:** Department of Pathology, Leiden University Medical Center, Albinusdreef 2, 2333 ZA Leiden, The Netherlands; Department of Clinical Epidemiology, Leiden University Medical Center, Albinusdreef 2, 2333 ZA Leiden, The Netherlands; Department of Clinical Oncology, Leiden University Medical Center, Albinusdreef 2, 2333 ZA Leiden, The Netherlands

**Keywords:** Immune contexture, Cervical cancer, Survival, Type I immunity, Prognosis

## Abstract

**Background:**

Deep invasion of the normal surrounding tissue by primary cervical cancers is a prognostic parameter for postoperative radiotherapy and relatively worse survival. However, patients with tumor-specific immunity in the blood at the time of surgery displayed a much better disease free survival. Here we analyzed if this was due to a more tumor-rejecting immune population in the tumor.

**Methods:**

Tumor sections from a group of 58 patients with deep normal tissue-invading cervical tumors were stained for the presence of immune cells (CD45), IFNγ-producing cells (Tbet) and regulatory T cells (Foxp3) by immunohistochemistry. The slides were scanned and both the tumor area and the infiltration of the differently stained immune cells were objectively quantified using computer software.

**Results:**

We found that an increased percentage of tumor occupied by CD45+ cells was strongly associated with an enhanced tumor-infiltration by Tbet+ cells and Foxp3+ cells. Furthermore, the area occupied by CD45+ immune cells, Tbet+ cells but not Foxp3+ cells within the tumor were, in addition to the lymph node status of patients, associated with a longer disease free survival and disease specific survival. Moreover, interaction analyses between these immune parameters and lymph node status indicated an independent prognostic effect of tumor infiltrating Tbet+ cells. This was confirmed in a multivariate Cox analysis.

**Conclusions:**

The area occupied by a preferentially type I oriented CD45+ cell infiltrate forms an independent prognostic factor for recurrence-free and disease-specific survival on top of the patient’s lymph node status.

**Electronic supplementary material:**

The online version of this article (doi:10.1186/s12967-015-0664-0) contains supplementary material, which is available to authorized users.

## Background

Cervical cancer (CxCa) develops as a result of an uncontrolled, persistent infection with a high-risk type of human papillomavirus (HPV), in particular types HPV16 and HPV18 [1]. The HPV genome encodes two oncoproteins, E6 and E7, which are constitutively expressed as they are required for the onset and maintenance of the malignant cellular phenotype [2]. These viral proteins form excellent targets for the immune system. Indeed HPV16-specific T cell responses are frequently detected in the blood of healthy individuals from which these cells can migrate upon antigenic challenge, showing that a successful defense against HPV is associated with systemically detectable T-cell responses against these viral antigens [3].

Previously, we have performed the largest prospective study on the HPV-specific immune response in relation to clinical prognostic factors in a group of 119 patients with HPV-induced CxCa. Proliferative T-cell reactivity against the HPV E6 and E7 oncoproteins was detected in the blood of about one-third of that group, most prominently in the group of patients with a tumor that deeply (≥15 mm) penetrated the healthy surrounding tissue [4]. Surprisingly, while deep invasion generally is associated with poor clinical outcome of CxCa [5], the patients displaying HPV-specific T cell reactivity in their blood had a significantly better 3-year disease free survival [4]. Animal studies have shown that the systemic spread of spontaneously-induced tumor-specific T cells requires local activation [6–8], suggesting that the detection of circulating tumor-specific T cells may represent a locally active immune population in the tumor microenvironment. This hypothesis is supported by a small study on HPV18-induced high grade cervical lesions where the number of lesion-infiltrating IFNγ-producing immune cells was found to be associated with the presence of HPV18-specific IFNγ-producing T cells in the blood [9].

Studies on the microenvironment of HPV-induced CxCa showed that decreased expression of the tumor-expressed T-cell ligands human leukocyte antigen class I and MHC class I chain-related molecule A had a negative impact on disease specific survival [10]. In contrast, the infiltration with high numbers of CD8+ T cells and relatively low numbers of CD4+ regulatory T cells (Tregs), as indicated by the expression of intra-nuclear FoxP3, is associated with better survival [10, 11]. Furthermore, a dense infiltration with tumor-infiltrating type 1 (M1) macrophages also was associated with improved disease-specific survival. Notably both the CD8+ to regulatory T-cell ratio and the number of M1 macrophages are independent prognostic factors for survival in CxCa [12]. The analyses of many different tumors revealed that strong infiltration with an activated type 1 T cell signature, reflected by the local expression of amongst others IFNγ/Tbet and granzymes, was associated with better prognostic outcome, better response to therapy or a higher chance to have a complete regression [13]. Expression of granzyme B has been detected in about 30 % of a small group of CxCa [14]. In addition, the expression of the programmed cell death receptor 1 was detected at the surface of about 50 % of the tumor-infiltrating CD4+ and CD8+ T cells [15] where it’s expression may reflect exhausted as well as activated T cells [16, 17]. Furthermore, the presence of M1 macrophages in a subgroup of patients suggest the local presence of IFNγ as this is required for the optimal polarization in the local tumor milieu [18]. Altogether this suggests that some CxCa patients have tumors with a more favorable immune microenvironment.

In order to study if some of the deep normal tissue-invading tumors are highly immunologically active and associated with a better clinical outcome, a group of 58 patients was studied with respect to CD45+ cell infiltration, the expression of Tbet as a measure for type 1 T cell signature and the expression of Foxp3 for regulatory T cells. We demonstrate that the presence of large tumor areas occupied by Tbet positive cells is associated with improved recurrence free survival and a lower risk of death by deeply invading cervical cancer.

## Methods

### Patient material

Formalin-fixed, paraffin-embedded (FFPE) CxCa specimens obtained from patients who underwent primary surgical treatment for CxCa between 1998 and 2006 with sufficient material available for analysis were obtained from the archives of the department of Pathology, Leiden University Medical Center (n = 58). Cervical adenosquamous carcinoma was discriminated from squamous cell carcinomas by using periodic acid Schiff plus and Alcian blue stainings in addition to a hematoxylin and eosin staining [19]. None of the patients had received preoperative anticancer therapy and follow-up data were obtained from patient medical records. Patient and tumor characteristics are listed in Table 1. Patient samples were handled according to the medical ethical guidelines described in the Code of Conduct for Proper Secondary Use of Human Tissue of the Dutch Federation of Biomedical Scientific Societies.

**Table 1:** Patient characteristics

	N	%
No. patients	58	
Mean age^a^	45	
Years (range)	25–87	
Figo stage^b^		
1b1, 1b2	47	81
2a, 2b	10	17
Unknown	1	
LN metastases^b^		
Yes	27	46
No	31	54
Tumor size^b^		
<4 cm	22	38
≥4 cm	36	62
Vasoinvasion^b^		
Yes	33	57
No	9	16
Unknown	16	27
Parametria involvement^b^		
Yes	9	16
No	37	64
Unknown	12	20

^a^At time of intervention

^b^At time of surgery

### Immunohistochemistry

Immunostainings were performed on 4 μm thick FFPE sections. Deparaffinized sections were treated with 0.3 % H_2_O_2_ in methanol for 20 min to block endogenous peroxidase activity. After rehydration, antigen retrieval was performed in Tris-ethylenediaminetetraacetic acid (EDTA) buffer (10 mM TRIS plus 1 mM EDTA pH 9.0) and mouse IgG1 anti-CD45 (clone 2B11+ PD7/26, Dako, Glostrup, Denmark) or mouse IgG1 anti-FoxP3 (clone 236A/E7, Abcam, Cambridge, UK) diluted in 1 % w/v bovine serum albumin (BSA) in phosphate buffered saline (PBS) was incubated at room temperature overnight. Subsequently, BrightVision poly-horseradish peroxidase (HRP)-anti mouse/rabbit/rat IgG (Immunologic, Duiven, The Netherlands) was incubated for 30 min and the HRP activity was visualized using 0.5 % 3,3′-diamino-benzidine-tetrahydrochloride (DAB) and 0.002 % H_2_O_2_ in TRIS–HCl. After antigen retrieval in citrate buffer pH6.0, rabbit anti-T-bet (H-210, Santa Cruz, TX, USA) diluted in 1 % BSA in PBS was incubated at room temperature overnight, followed by incubation with DAB+ (Dako). Sections were counterstained with hematoxylin and slides were mounted using CV Mount (Leica Microsystems).

### Image analysis

**Fig. 1:**
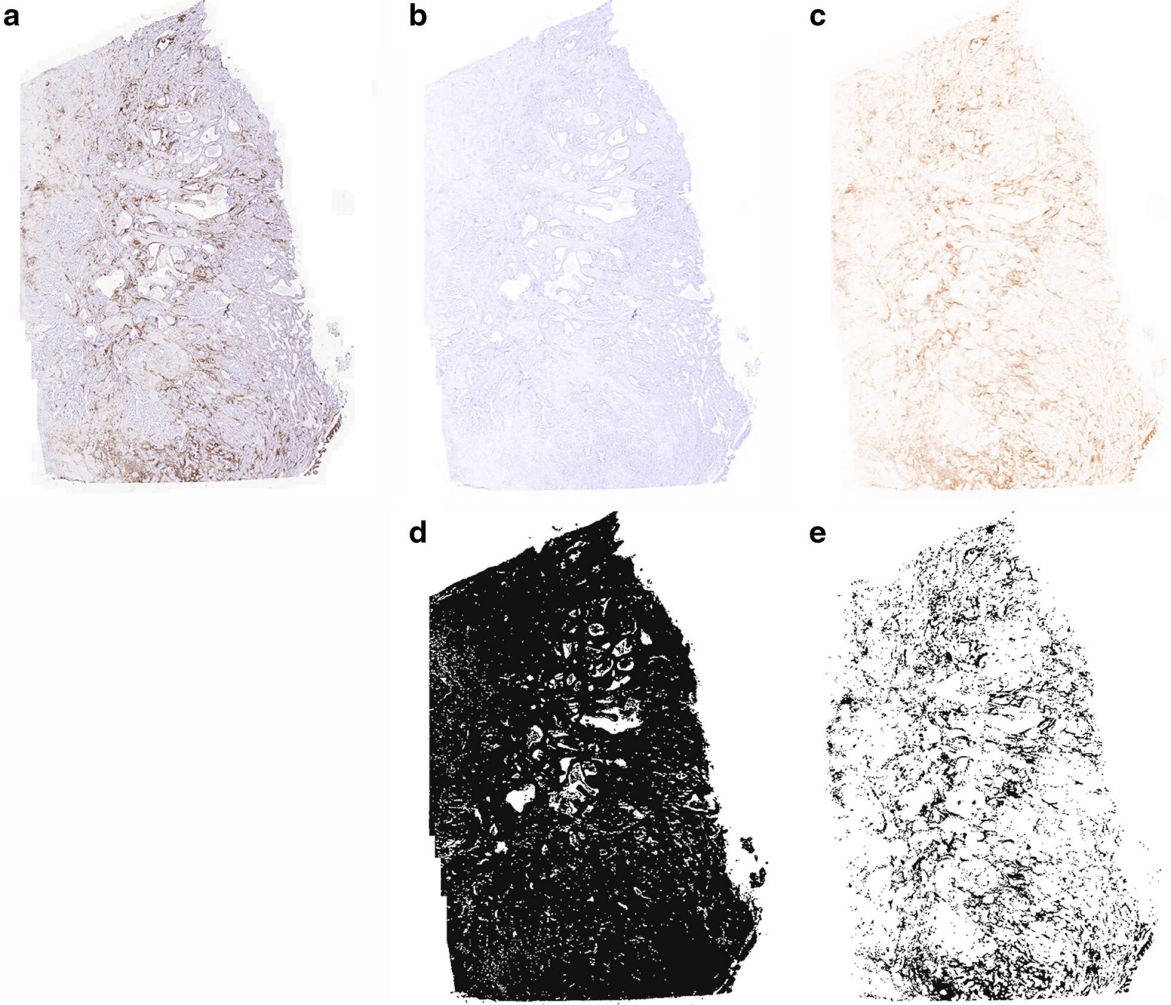
Schematic representation of the automated quantitative method used. A representative image of a scanned slide (**a**). After deconvoluting the haematoxylin (**b**) and DAB (**c**) colors, the total tissue area was selected using a 100 % threshold for the haematoxylin staining (**d**). Then the DAB positive area was determined (**e**). The number of pixels in image **e** was divided by the number of pixels in image **d** and multiplied by 100 to calculate the tumor percentage expressing DAB

Slides were scanned with a Panoramic Midi automated slide scanner (3DHISTECH, Budapest, Hungary) with a 20× objective. From the scanned slides the tumor area was selected at the same magnification. The images were analyzed with ImageJ (1.47v; developed by Wayne Rasband, National Institute of Health, MD, USA). Colors were separated using the color deconvolution plugin Haematoxylin and DAB (H DAB), as shown in Fig. 1. First, the total tissue area was selected with a 100 % threshold of the haematoxylin staining (Fig. 1b, d). Subsequently, the area occupied by the DAB signal was selected using an appropriate threshold level (Fig. 1c, e). Both areas were recorded as a number of pixels. The DAB area divided by the total tissue area (100×) provides the percentage of tissue occupied by DAB positive cells. Representative images of the staining at a 200× magnification were obtained by using a 40× objective.

### Statistics

Statistical analyses were performed using SPSS version 20.0 (IBM Corp., Armonk, USA). Correlation between the area occupied by the different types of DAB-positive cells and clinical variables were tested with Pearson’s correlation, paired t test and Wilcoxon Mann–Whitney tests, as appropriate. Correlation between clinical variables or immune parameters and disease-specific or recurrence-free survival was calculated by the Kaplan–Meier method and analyzed by the log-rank test. Univariate and multivariate Cox proportional hazards models were used to determine the hazard ratio (HR) that represents the relative risk of death among patients in the different indicated groups. Two-sided *P* values of <0.05 were judged to be significant.

## Results

### Patient characteristics

In order to study if immunologically active tumors were associated with better clinical outcome a group of 58 women with Figo stage 1b to 2b CxCa was selected based on a surrounding tissue infiltration depth of 15 mm or more. Patients with Figo stages 1a were not selected because they have a near to 100 % survival rate after surgery, whereas patients with Figo stages 3 and 4 were not selected since they are treated by primary chemoradiation. Patient characteristics are shown in Table 1. The mean age of the 58 patients was 45 years (range 25–87). The infiltration depth ranged from 15 to 95 mm (mean 25). The average tumor size was 50 mm (range 20–130) and lymph node metastases were found in 27 patients. The great majority was squamous cell carcinoma and treated with postoperative radiotherapy. The average total follow up time was 57 months (range 2.2–147).

### The tumor area occupied by immune cells increases with a stronger co-infiltration of Tbet+ and Foxp3+ cells

**Fig. 2:**
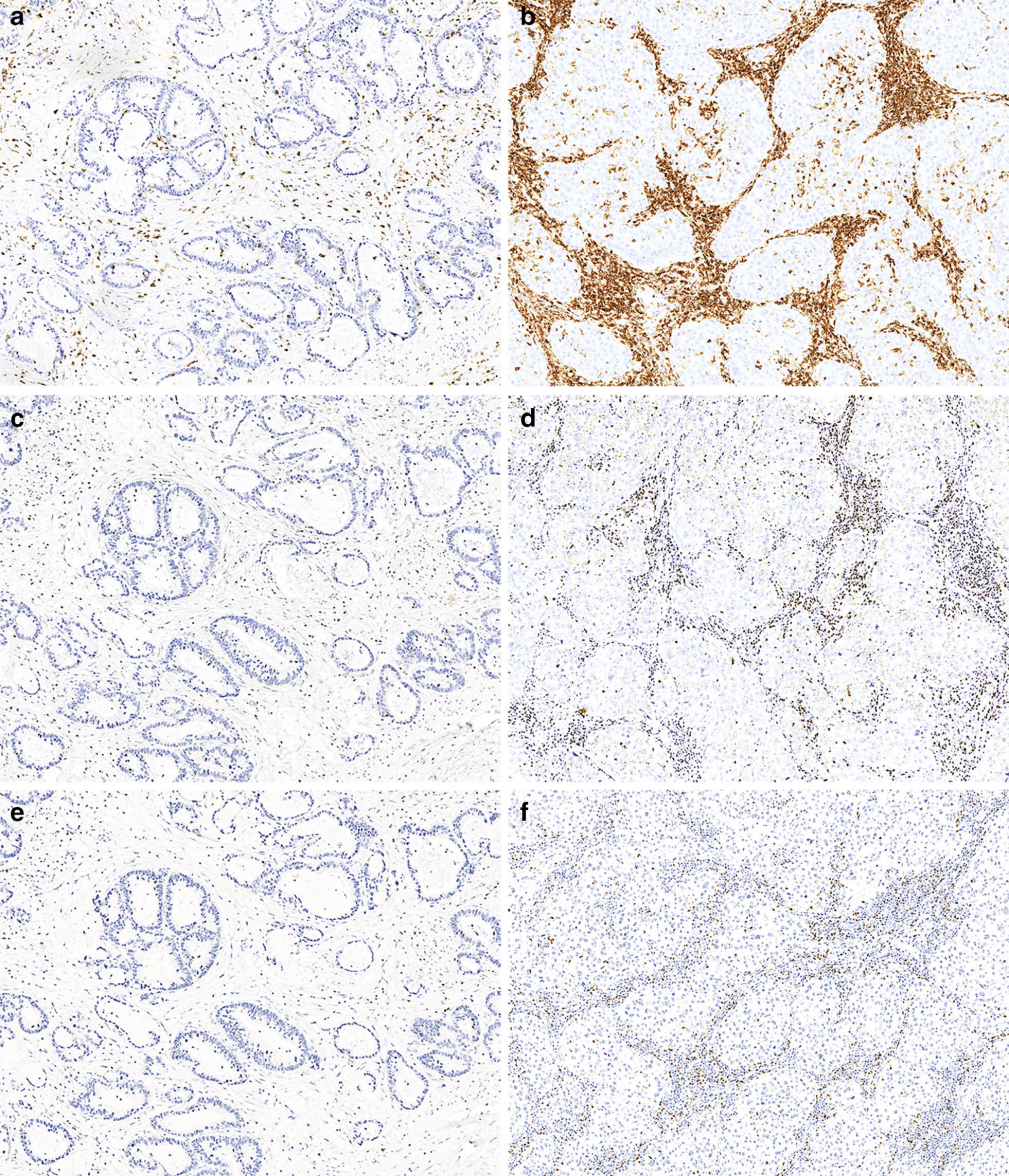
Immunohistochemical staining for CD45, T-bet and FoxP3. Representative images of immunohistochemical stainings for CD45 (**a**, **b**), T-bet (**c**, **d**) and FoxP3 (**e**, **f**) are shown. On the *left* is a tumor sample with low lymphocyte frequencies and on the *right* a tumor sample with high lymphocyte frequencies (**b**, **d**, **f**). Images were obtained at a ×200 magnification

We used an objective quantitative automated method to analyze immune cell infiltration of tumors (Fig. 1). In 52/58 cases tumor sections could be stained for the presence of CD45+ cells, the presence of Tbet+ cells, as a measure for IFNγ-producing cells, and the presence of Foxp3+ cells for regulatory T cells. Analyses of all tumors using Image J revealed that the mean number of pixels representing tumor infiltrating CD45+ cells was high (5672 per HPF (20×); range 1613–8572) and that these cells occupied on average 17 % of the tumor area (range 0.6–58 %). The mean number of pixels representing Tbet+ cells was 302 per HPF (range 9–3069) and that of Foxp3+ cells was 142 per HPF (range 6–481). Figure 2 shows an example of a tumor with high numbers of infiltrating immune cells and a tumor with low numbers of infiltrating immune cells. Statistical analyses showed that overall (p < 0.0005; Mann–Whitney) but also in each sample (p < 0.0005; paired sample t test) the pixel count for tumor-infiltrating CD45+ cells was higher than that of Tbet+ cells and that of Foxp3+ cells, indicating that these two functional cell types were not the only types of immune cells infiltrating the tumor. An increase in the total pixel count of tumor-infiltrating immune cells was often reflected in higher pixel count for Tbet+ (r = 0.267, p = 0.061; Pearson) and Foxp3+ cells (r = 0.414, p = 0.005; Pearson). There was a strong positive correlation between the pixel count of Tbet+ and Foxp3+ cells (r = 0.539, p < 0.0005; Pearson) indicating that these cell types frequently co-infiltrated the tumor. However, the pixel count for tumor-infiltrating Tbet+ cells was generally (p < 0.0005; Mann–Whitney) but also in each sample (p = 0.011; paired sample t test) higher than that of Foxp3+ cells. Interestingly, the pixel count for Tbet+ and Foxp3+ cells positively correlated with the total tumor area occupied by immune cells (r = 0.596 and r = 0.681, respectively, p < 0.0005; Pearson), suggesting that especially the infiltration with T cells resulted in a larger immune to tumor cell ratio.

### A larger immune infiltrate in patients with circulating proliferative HPV-specific T cells

Among the group of 58 patients, 17 were previously tested for the presence of HPV-specific T cell reactivity in the blood [4], 10 of whom were positive. The mean area occupied by CD45+ cells in the corresponding tumors was larger in patients with a proliferative response than in the tumors of patients without such reactivity (Table 2). Similarly, the pixel count of tumor-infiltrating Tbet+ cells was twofold higher in the group with a circulating HPV-specific T cell response, whereas the mean pixel count of Tregs or total cells did not differ (Table 2). Although the data were not statistically significant in this small patient group, the results suggest that the detection of a proliferative response to HPV in the blood may be associated to a more active local immune response.

**Table 2:** Differences in immune cell infiltration between groups

	No		Yes		*p* value^a^
	Mean	SEM	Mean	SEM	
HPV-specific T cells in blood^b^					
Area of CD45 cells (%)	9.76	2.51	15.47	2.93	0.203
Total cell pixel count	5378	619	5678	377	0.667
Tbet+ cells pixel count	244	72.0	434	185	0.485
Foxp3+ cells pixel count	125	25.9	142	24.2	0.648
LN metastases present					
Area of CD45 cells (%)^c^	20	3.3	14	2.0	0.119
Total cell pixel count^d^	5686	339	5655	183	0.942
Tbet+ cells pixel count^d^	405	118	174	42	0.097
Foxp3+ cells pixel count^d^	163	27.5	124	16.8	0.217
Tumor size ≥40 mm					
Area of CD45 cells (%)	20	3.4	16	2.5	0.346
Total cell pixel count	5806	440	5590	189	0.656
Tbet+ cells pixel count	488	162	189	40.6	0.087
Foxp3+ cells pixel count	170	27.4	124	18.7	0.152
Area CD45 cells (%) ≥ median					
Total cell pixel count	5460	264	5919	279	0.239
Tbet+ cells pixel count	114	27.9	328	55.0	0.002
Foxp3+ cells pixel count	80.6	12.5	196	25.9	0.000

^a^Differences in means were assessed by independent sample t test, p < 0.05 is significant

^b^Within the current study group 17 patients have been tested for the presence of circulating HPV-specific T cells, 10 of them were negative [4]

^c^The area occupied by CD45 cells within the tumor was calculated as a percentage

^d^The pixel count of cells per high power field (20×) was analysed in 4 different fields

### A large area with Tbet associated immune cell infiltrate prevents recurrences and improves disease specific survival

**Fig. 3:**
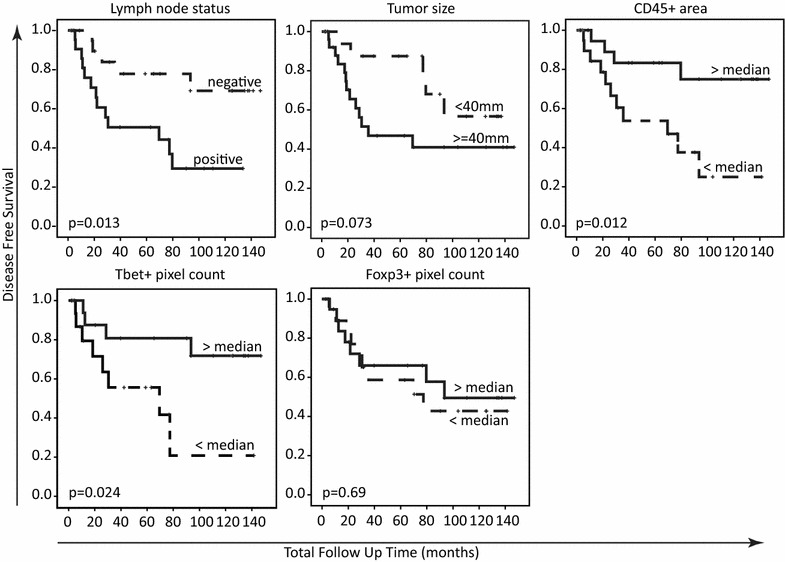
Kaplan–Meier disease free survival curves. The patient group was divided based on two clinicopathological parameters (lymph node status or tumor size) or based on the three tested immune factors, the tumor area occupied by CD45+ cells, and the Tbet+ and Foxp3+ pixel count representing number of Tbet+ and Foxp3+ cells infiltrating the tumor, respectively. A Log-Rank test was used to determine whether the difference in disease free survival was significant

The known prognostic factors in CxCa are lymph node metastases (present or absent), tumor size (< or ≥40 mm) and the invasion depth of the tumor in the surrounding cervical tissue (< or ≥15 mm) [5]. When the disease-free survival (DFS) and the disease specific survival (DSS) curves of the CxCa patients were plotted based on these factors, the patients with lymph node metastases (p = 0.013; Log-Rank) and a tumor ≥40 mm (p = 0.073; Log-Rank) were more prone to develop a recurrence (Fig. 3). While there were no differences with respect to the area occupied by immune cells, the pixel count of CD45+ cells or Foxp3+ cells, the pixel count of tumor-infiltrating Tbet+ cells was doubled in patients without lymph node metastases and with smaller tumors (Table 2). In addition, the DSS of patients was associated with the lymph node status (p = 0.043; Log-Rank) but not with tumor size (p = 0.132; Log-Rank).

Then the DFS and DSS of the patients were plotted based on the four immune factors. The area occupied by CD45+ immune cells within the tumor was associated with a longer DFS (p = 0.012; Log-Rank; Fig. 3) and DSS (p = 0.016; Log-Rank). Notably, the larger CD45+ cell occupied areas comprised about threefold more Tbet+ cells and twofold more Foxp3+ cells than the smaller areas (Table 2) while the total pixel count of CD45+ cells increased marginally. This suggests that a relatively larger area of CD45+ cells within the tumor is the result of a more pronounced T-cell infiltration, irrespective of their functional phenotype, as no differences in the relative proportion of Tbet+ and Foxp3+ were found between tumors with relatively larger or smaller CD45+ cell areas (p = 0.947; Pearson). Next the group of patients was divided on the basis of infiltration with Tbet+ cells and Foxp3+ cells. Infiltration with Foxp3+ cells showed no association with DFS (p = 0.69; Log-Rank) or DSS (p = 0.24; Log-Rank). Similar results were obtained when Foxp3+ cell infiltration was analyzed as a ratio of the total numbers of CD45+ cells. A higher pixel count of tumor-infiltrating Tbet+ cells, however, was positively associated with DFS (p = 0.024; Log-Rank; Fig. 3) and DSS (p = 0.009; Log-Rank). Also the ratio between the pixel count of Tbet+ cells and CD45+ cells showed a positive association with DFS (p = 0.029; Log-Rank) and DSS (p = 0.019; Log-Rank), indicating that if more tumor-infiltrating immune cells display an IFNγ-associated phenotype, the risk of a recurrence or death due to CxCa is reduced. There was no difference in DFS or DSS when patients were divided according to the ratio of Foxp3+ cells and Tbet+ cells, indicating that the impact of Tbet+ cells does not depend on the co-infiltrating Tregs. This analysis confirms that the presence of a large area with IFNγ-positive cells (Tbet+) is associated with less recurrences and longer disease specific survival.

### Immune infiltration is an independent prognostic factor for survival

**Fig. 4:**
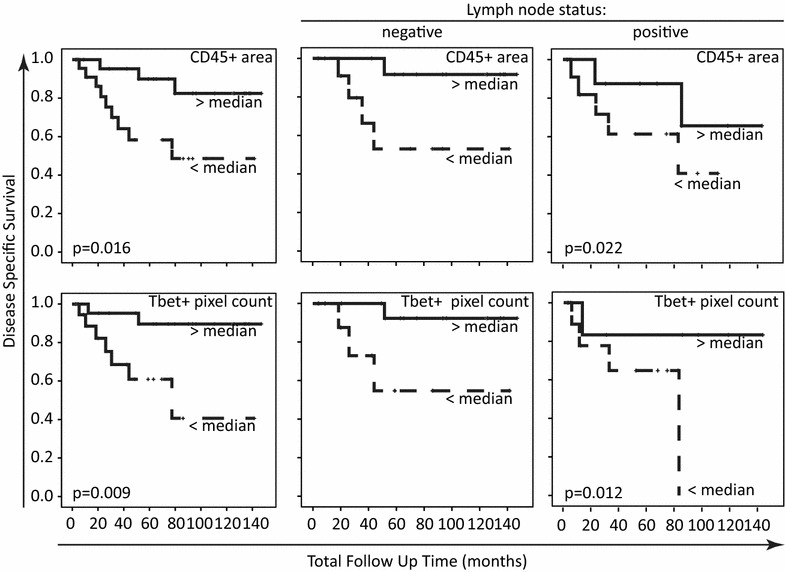
Kaplan–Meier disease specific survival curves. The patient group was divided based on either the occupancy of the tumor area by CD45+ cells, or based on the Tbet+ pixel count representing the number of Tbet+ cells infiltrating. Disease specific survival was plotted (*left* two graphs). In addition, these two parameters were analyzed in the context of the lymph node status of the patient group. Log-Rank analysis was used to determine the statistical significance of the difference in disease specific survival

Since the lymph node status is strongly associated with survival, we analyzed the DFS and DSS outcomes of the area occupied by CD45+ cells and the infiltration with Tbet+ cells in this context. Clearly the CD45 area (p = 0.016; Log-Rank; Fig. 4) and the pixel count for Tbet+ cells (p = 0.009; Log-Rank; Fig. 4) had a positive effect on DSS, irrespectively of the lymph node status. A similar observation was made with respect to the area of CD45+ cells (p = 0.039; Log-Rank) and Tbet+ cells (p = 0.083; Log-Rank) with DFS. An interaction analysis (Additional file 1: Figure S1) based on the four categories formed by patients with absence or presence of lymph node metastases and a high or low pixel count for Tbet+ cells confirmed the positive effect of Tbet+ cells for DFS (p = 0.032; Log-Rank) and DSS (p = 0.062; Log-Rank). The same was observed for the four groups based on lymph node status and a large or small area of CD45+ cells, showing that the area occupied by immune cells was a strong factor with respect to DFS (p = 0.014; Log-Rank) and DSS (p = 0.083; Log-Rank).

Finally, a multivariate Cox analysis was performed in which the area occupied by CD45+ cells or the infiltration with Tbet+ cells were used as covariate besides lymph node status, the latter being the strongest clinical pathological marker. Both the lymph node status and the immune infiltrate displayed a strong effect on DFS and DSS. Irrespective of the lymph node status, patients of whom the tumor displayed a relatively larger area with CD45+ cells had a lower risk for recurrences (HR 0.300; CI 0.093–0.964; p = 0.043) and a lower risk to die of CxCa (HR 0.245; CI 0.065–0.924; p = 0.038). Also, tumor-infiltration with higher pixel count for Tbet+ cells corresponded with a lower risk for recurrences (HR 0.306; CI 0.084–1.109; p = 0.071) or to die of CxCa (HR 0.175; CI 0.034–0.887; p = 0.035). Thus, the area occupied by CD45+ cells or the infiltration with Tbet+ cells form two independent prognostic factors for recurrence-free and disease-specific survival on top of the patient’s lymph node status.

## Discussion

The presence of lymph node metastases, the tumor size and the deep penetration of the healthy surrounding tissue by CxCa generally is associated with poor outcome [5]. In a previous study we detected systemic anti-tumor responses predominantly in patients with deep invading tumors and this was associated with a better disease free survival [4]. Here we zoomed in on the group of patients with deeply invading tumors. To obtain objective results, computer software was used to automatically quantify CD45+, Tbet+, or FoxP3+ cell infiltration. Calculation of the percentage of tumor tissue occupied by CD45+ immune cells revealed a wide range within this group. Importantly, the area occupied by immune cells showed a positive correlation with recurrence free and disease specific survival. While the majority of the tumor infiltrating immune cells did not express Tbet or Foxp3, an increased immune percentage of tumor occupied by CD45+ cells was associated with the enhanced tumor-infiltration by Tbet+ cells and Foxp3+ cells. Notably, the mean and range of the number of infiltrating cells differed considerably between the subtypes being the highest for CD45+ cells, followed by Tbet+ cells and the lowest for Foxp3+ cells. Furthermore, the correlation coefficients (*r*) in the Spearman’s rank test between the different cells ranged from 0.267 to 0.539. This indicates that while there is a tendency for different types of immune cells to simultaneously infiltrate the tumor this is not at a one-to-one basis and as such correlations between immune cell infiltration and clinical outcome not necessary overlap. Based on our earlier work a substantial amount of the infiltrating cells comprise different subsets of myeloid cells [12]. The Tbet+ cells are most likely IFNγ-producing T cells as NK cells have never been found in large numbers in CxCa [10]. Infiltration with Tbet+ cells but not Foxp3+ cells was also positively associated with DFS and DSS, indicating that the type 1 immune orientation of the infiltrate, rather than the overall number of immune cells is an important predictor for the response to subsequent therapy. There are also indications that the dense infiltration with Tbet+ cells is associated with better clinical outcome in HPV-induced premalignant lesions [9, 17]. These data fit well with the role of IFNγ producing lymphocytes in the prevention and control of cancer cells in mice [20] as well as with similar observations made on type 1 T cells and improved response to therapy in ovarian [21], colorectal [22], renal cell [23] and lung cancer [24].

Of the clinical pathological markers the lymph node status is an important predictor for the DFS and to a lesser extent for the DSS of patients with deep invading tumors. The tumors of patients without lymph node metastases on average had the largest area occupied with CD45+ immune cells and displayed a stronger infiltration with Tbet+ cells when compared to the tumors of patients with lymph node metastases. However, both immune parameters showed a significant impact on DFS and DSS irrespective of the lymph node status and these effects were retained in a multivariate Cox analysis.

Based on a study showing that in a wide variety of cancers the infiltrating Foxp3+ cells were functional Tregs [25], making it likely that the Foxp3+ cells detected in our study reflect Tregs. A high infiltration with Tregs has been associated with poor clinical outcome in ovarian cancer [26] and breast cancer [27] but we did not find such a direct effect of the infiltrating Tregs in our study. Other studies on hepatocellular cancer [28] and CxCa [10] did not find a direct relation between Treg infiltration and clinical outcome, instead the ratio between CD8+ T cells and Tregs had prognostic value. However, the ratio between Tbet+ cells and Tregs did not change the DFS or DSS curves when compared to Tbet as a single marker. Therefore, the clinical outcome of patients with deeply invading cervical tumors is not influenced by the Foxp3+ cells, which is also the case in glioma [29] and cutaneous melanoma [30].

The observation that the percentage of tumor area occupied by immune cells—as measured by a simple objective quantitative automated method—showed a positive correlation with recurrence free and disease specific survival opens up possibilities to use this technique in clinical management of the patients. One can envision that absolute cut-offs for the percentage of tumor occupied by immune cells can be defined to identify patients who do not benefit from the current standard therapy (low % occupied) or to select patients most likely to have a good clinical response upon immunotherapy. This will require validation of our conclusions in a larger dataset similar to what is done for the immunoscore in colorectal cancer [31].

## Conclusions

Our previous study identified a subgroup of patients with cervical tumors deeply invading the surrounding normal tissue, who unexpectedly had a better outcome when they displayed an effector T-cell response against their tumor as evidenced by the presence of higher percentages of tumor-specific effector T cells and Tregs circulating in the blood [4]. By the use of an objective quantitative automated method that can readily be integrated in Pathology labs we confirmed that such a subgroup of patients with deeply tissue invading tumors displaying active tumor-immunity exists and that a strong type 1 effector response within these tumors forms an important prognostic factor for clinical outcome.

## Additional file

**Additional file 1: Figure S1.** Interaction analysis of lymph node status and immune parameters. The patient group was divided based on the four categories formed by patients with absence/presence of lymph node metastases and a large/small area of CD45+ cells (left graphs) or a high/low pixel count for Tbet+ cells (right graphs). For these groups the Kaplan–Meier curves for disease specific survival (top row) and the recurrence free survival (bottom row) were plotted. Log-Rank analysis was used to determine the statistical significance of the difference in survival.
