# Supplementary figures and images for: The tumor area occupied by Tbet+ cells in deeply invading cervical cancer predicts clinical outcome
Source: J Transl Med. 2015 Sep 10;13:295. doi: 10.1186/s12967-015-0664-0 (PMC4566330; doi:10.1186/s12967-015-0664-0)

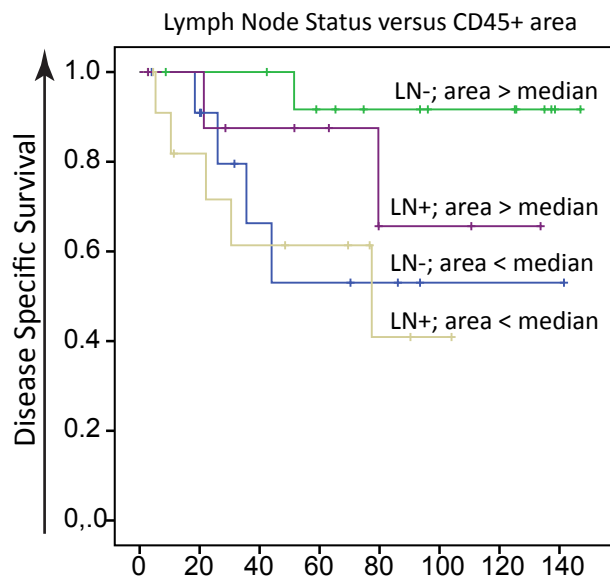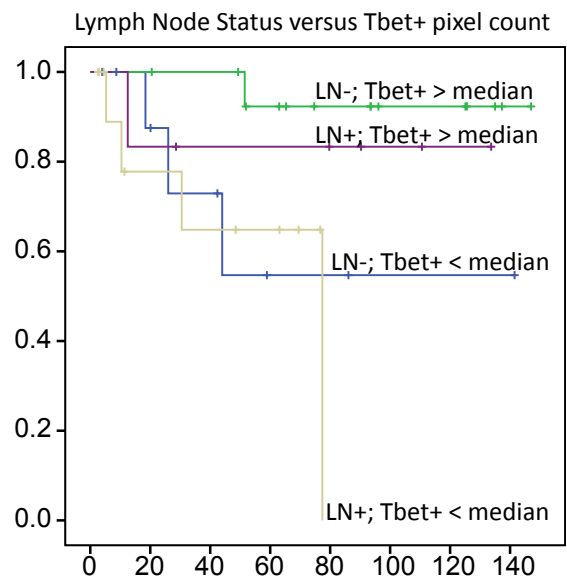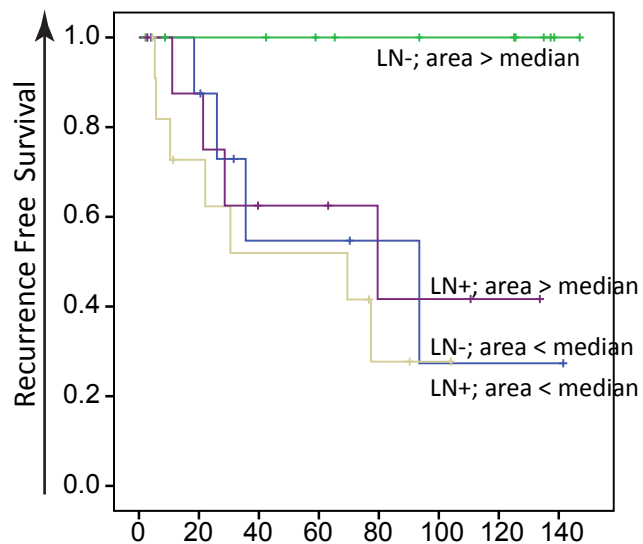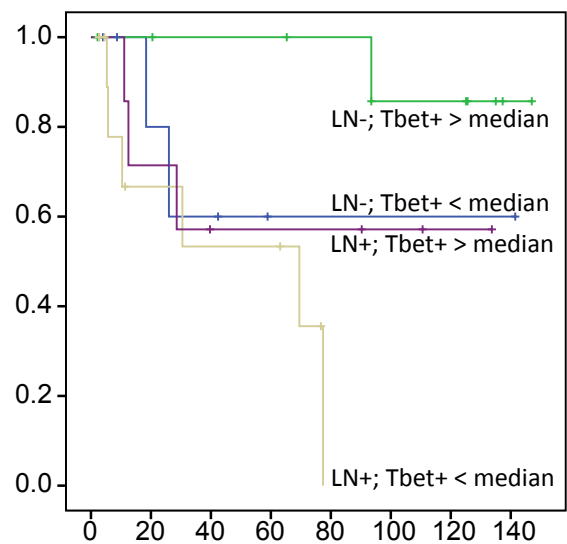

Total Follow Up Time (months)

Supplement: Supplementary file 1 — Additional file 1: Figure S1. Interaction analysis of lymph node status and immune parameters. The patient group was divided based on the four categories formed by patients with absence/presence of lymph node metastases and a large/small area of CD45+ cells (left graphs) or a high/low pixel count for Tbet+ cells (right graphs). For these groups the Kaplan–Meier curves for disease specific survival (top row) and the recurrence free survival (bottom row) were plotted. Log-Rank analysis was used to determine the statistical significance of the difference in survival. [file 12967_2015_664_MOESM1_ESM.pdf]
